# Synthesis and Characterization of Dual-Functionalized Core-Shell Fluorescent Microspheres for Bioconjugation and Cellular Delivery

**DOI:** 10.1371/journal.pone.0050713

**Published:** 2013-03-19

**Authors:** Jonathan M. Behrendt, David Nagel, Evita Chundoo, Lois M. Alexander, Damien Dupin, Anna V. Hine, Mark Bradley, Andrew J. Sutherland

**Affiliations:** 1 Chemical Engineering & Applied Chemistry, School of Engineering & Applied Science, Aston University, Birmingham, United Kingdom; 2 School of Life & Health Sciences, Aston University, Birmingham, United Kingdom; 3 School of Chemistry, Edinburgh University, Edinburgh, United Kingdom; 4 Department of Chemistry, Dainton Building, the University of Sheffield, Brook Hill, Sheffield, United Kingdom; Brandeis University, United States of America

## Abstract

The efficient transport of micron-sized beads into cells, via a non-endocytosis mediated mechanism, has only recently been described. As such there is considerable scope for optimization and exploitation of this procedure to enable imaging and sensing applications to be realized. Herein, we report the design, synthesis and characterization of fluorescent microsphere-based cellular delivery agents that can also carry biological cargoes. These core-shell polymer microspheres possess two distinct chemical environments; the core is hydrophobic and can be labeled with fluorescent dye, to permit visual tracking of the microsphere during and after cellular delivery, whilst the outer shell renders the external surfaces of the microspheres hydrophilic, thus facilitating both bioconjugation and cellular compatibility. Cross-linked core particles were prepared in a dispersion polymerization reaction employing styrene, divinylbenzene and a thiol-functionalized co-monomer. These core particles were then shelled in a seeded emulsion polymerization reaction, employing styrene, divinylbenzene and methacrylic acid, to generate orthogonally functionalized core-shell microspheres which were internally labeled *via* the core thiol moieties through reaction with a thiol reactive dye (DY630-maleimide). Following internal labeling, bioconjugation of green fluorescent protein (GFP) to their carboxyl-functionalized surfaces was successfully accomplished using standard coupling protocols. The resultant dual-labeled microspheres were visualized by both of the fully resolvable fluorescence emissions of their cores (DY630) and shells (GFP). *In vitro* cellular uptake of these microspheres by HeLa cells was demonstrated conventionally by fluorescence-based flow cytometry, whilst MTT assays demonstrated that 92% of HeLa cells remained viable after uptake. Due to their size and surface functionalities, these far-red-labeled microspheres are ideal candidates for *in vitro*, cellular delivery of proteins.

## Introduction

The cellular membranes of eukaryotes are remarkable not only for their ability to partition the cell from its external environment but also to enable selective transport of materials into and out of the cell. Indeed from an evolutionary perspective, it is widely accepted that organelles such as mitochondria evolved from bacteria that were originally engulfed by cells in a mutually beneficial process termed endosymbiosis [1], [2]. The inherent propensity of eukaryotic cell membranes to enable transport of such large entities into cells presumably provided their cellular ancestors with some kind of advantage over cells that lacked such ability. In recent times researchers have been able to hijack this proclivity to enable particles and particles bearing relevant molecular cargoes to be imported directly into cells.

It is well known that cells readily take up nanoparticles. The types of nanoparticle that are taken into cells are myriad and include inorganic-based materials such as gold nanoparticles [3], [4], silica nanoparticles [5], quantum dots [6] etc. Alternatively the particles can be organic-based; notable recent examples here include polymeric nanoparticles based on self-assembled π-conjugated oligomers [7], dendrimers [8], polymer-beads [9] and liposomal preparations [10]. Advantages of both organic and inorganic components are often combined in hybrid systems where the advantages conferred by each component are shared to generate a whole that is greater than the sum of the individual parts [11]. Regardless of composition, the nanoparticles themselves may be used directly either i) for bioimaging/biosensing applications or ii) as intracellular delivery vectors. For both types of application it is often desirable to conjugate molecules, such as proteins, to the outer, solvent accessible surfaces of the particles [12]. For delivery applications it is often desirable to develop a system that is capable of delivering a molecular cargo with concomitant imaging of the delivery vector [13]. Such a system, although more complex to establish, allows key information, such as which cells have received the molecular cargo, to be readily determined.

Despite the numerous examples describing the use of nanoparticles as imaging/delivery agents there are still inherent drawbacks associated with their use. It is known that uptake of such small particles damages the cells [14] and this is especially the case when the nanoparticles possess high aspect ratios (i.e. they are long and narrow in shape) [15].

Moreover, small nanoparticles (<100 nm) are generally taken up by endocytosis. This exposes them and critically, any cargoes that they bear, to the degrading environments of endosomes/lysosomes. Certainly methods exist to help ameliorate this problem by promoting endosomal/lysosomal escape (e.g. by coating nanoparticles with polymer layers that act as ‘proton sponges’ [16], [17]) but it is clearly advantageous to avoid this uptake pathway altogether.

It is perhaps not so well known but cells are also readily capable of taking up much larger entities than nanoparticles. Presumably this capability results directly from such rapacious cells being selected for early on in their evolutionary pathway. In the laboratory, the cellular uptake of microspheres by macrophages was first observed thirty years ago [18]. Uptake in this instance was thought to occur, perhaps unsurprizingly given the cell type, by a phagocytosis-mediated process. More recently, it has been shown that polystyrene microspheres, with diameters up to 2 µm (about the size of a typical prokaryotic cell) can be efficiently transported into a range of cell types. It has been shown that the mechanism of uptake, in these non-phagocytic cells, is a passive but rapid process and is not endocytosis-mediated [19]. Thus microsphere-mediated delivery offers an advantage over nanoparticle-based delivery by preventing any exposure of biomolecular cargoes to the harsh environments of lysosomes and endosomes.

We and others term the internalization process, whereby such large microspheres are taken up by cells, as “beadfection” [19]. To date the microspheres used in beadfection have been polystyrene-based and as such are generated readily by using commonly employed emulsion and dispersion polymerization reactions. By including functional co-monomers in these polymerization reactions it is possible to generate microspheres with a wide range of functional groups, including amines, carboxylic acids, thiols and aldehydes. Such functional groups render the microspheres amenable to many possible covalent attachment strategies, which in turn enables conjugation of biologically relevant molecular cargoes [20]–[22]. For example, amino-functionalized polystyrene microspheres have been labeled with fluorescein and subsequently shown to readily enter a wide variety of cell lines with high efficiency and negligible toxicity. Microspheres of this type have been used to measure intracellular pH at the single cell level [23]. Similarly, attachment of indo-1 and fura-2 dyes to amino-functionalized microspheres has given rise to microsphere-based calcium sensors that enable intracellular calcium levels to be determined. Moreover these materials can give measurable signals which are not diluted within the cell thus allowing them to be used for a prolonged and accurate analysis [24]. It has also been demonstrated that fluorescence activated cell sorting (FACS) can be used as a basis for sorting cells beadfected with fluorescently-labelled microspheres [25]. Such bioimaging/sensing applications have been complemented recently by reports that beadfection may also be employed to transport both functional proteins [26] and active short interfering RNA (siRNA) [27] into cells. To date, the microspheres that have been used in beadfection studies have been polystyrene-based with a single surface functionality (e.g. amino groups). Where it has been desirable to both attach a biological cargo to the microspheres and to attach a fluorescent label to monitor the beadfection process, elaborate branched linker molecules have been constructed at the microsphere surface, which require multiple synthetic steps and protective group strategies.[27] We therefore wished to revaluate the microspheres used in beadfection and develop new materials which would enable simultaneous cellular imaging and delivery of biomolecules using microspheres to become more generally applicable.

This study reports the synthesis and evaluation of novel microspheres designed specifically for beadfection, to make the process more generally applicable for *in vitro* use. These microspheres are of the appropriate size (ca 1–2 µm), hydrophilic to prevent aggregation in aqueous media, and are doubly-functionalized to permit both facile bioconjugation and concomitant fluorescent visualization. Finally, FACS studies demonstrate that these microspheres are internalized by HeLa cells.

## Results and Discussion

### Microsphere design

This study aimed to develop cellular delivery agents, based on microspheres, that can both carry biologically relevant molecular cargo(es) and also permit imaging of the delivery process and sorting of cells which have received the biological cargo from those which have not been ‘beadfected’. In general, protein immobilization onto microspheres results in the outer surfaces only(≤15% of the volume of the microsphere) being derivatized with protein, typically because the protein is too large to diffuse into the microspheres [28]. Accordingly, we elected to construct orthogonally-functionalized microspheres that possess hydrophobic thiol-functionalized cores (to enable labeling with thiol-reactive dyes for bead visualization both pre- and post-beadfection) and an outer, carboxyl-functionalized shell to enable protein immobilization and render the microspheres hydrophilic, for cellular compatibility. This approach is simple yet highly effective due to the specific nature of thiol-based ‘click’ reactions. As such a thiol reactive dye can be reacted with the microspheres in the presence of the shell carboxyl groups without need for laborious protecting group strategies.

### Microsphere synthesis

**Figure 1:**
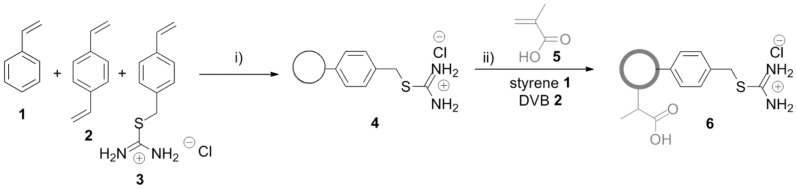
Synthesis of core-shell microspheres. Reaction conditions: i) PVP 58k, azobis*iso*butyronitrile (AIBN), hexadecane, 70°C, 16 h ii) AIBN, hexadecane, SDS(aq), RT 70°C, 5 h.

To form a hydrophobic core, styrene **1**, divinylbenzene (DVB) **2** and the thiouronium-containing monomer (4-VBTU) **3** were co-polymerized in a dispersion polymerization reaction, using methodology we have described previously [29], to form thiouronium-functionalized microspheres **4**. To add a hydrophilic shell, we adapted the methodology developed by Bradley *et.* al [30] and Kobayashi and Senna [31], who have previously described the growth of a uniform polymer shell composed of styrene and a functional vinyl co-monomer around seed particles. Specifically, microspheres **4** were readily shelled with a cross-linked, co-polymer layer comprizing styrene, divinylbenzene and methacrylic acid (MAA) **5**, to form core-shell microspheres **6**. Inclusion of styrene **1** and DVB **2** in the shell was intended to confer sufficient hydrophobic character to the shell such that, when swollen in suitable solvents such as DMF and toluene, apolar dyes could traverse the shell to reach the hydrophobic core. In addition, crosslinking with DVB would confer physical stability to the shell, so enabling prolonged incubations and subsequent imaging (Figure 1).

### Microsphere characterization

**Figure 2:**
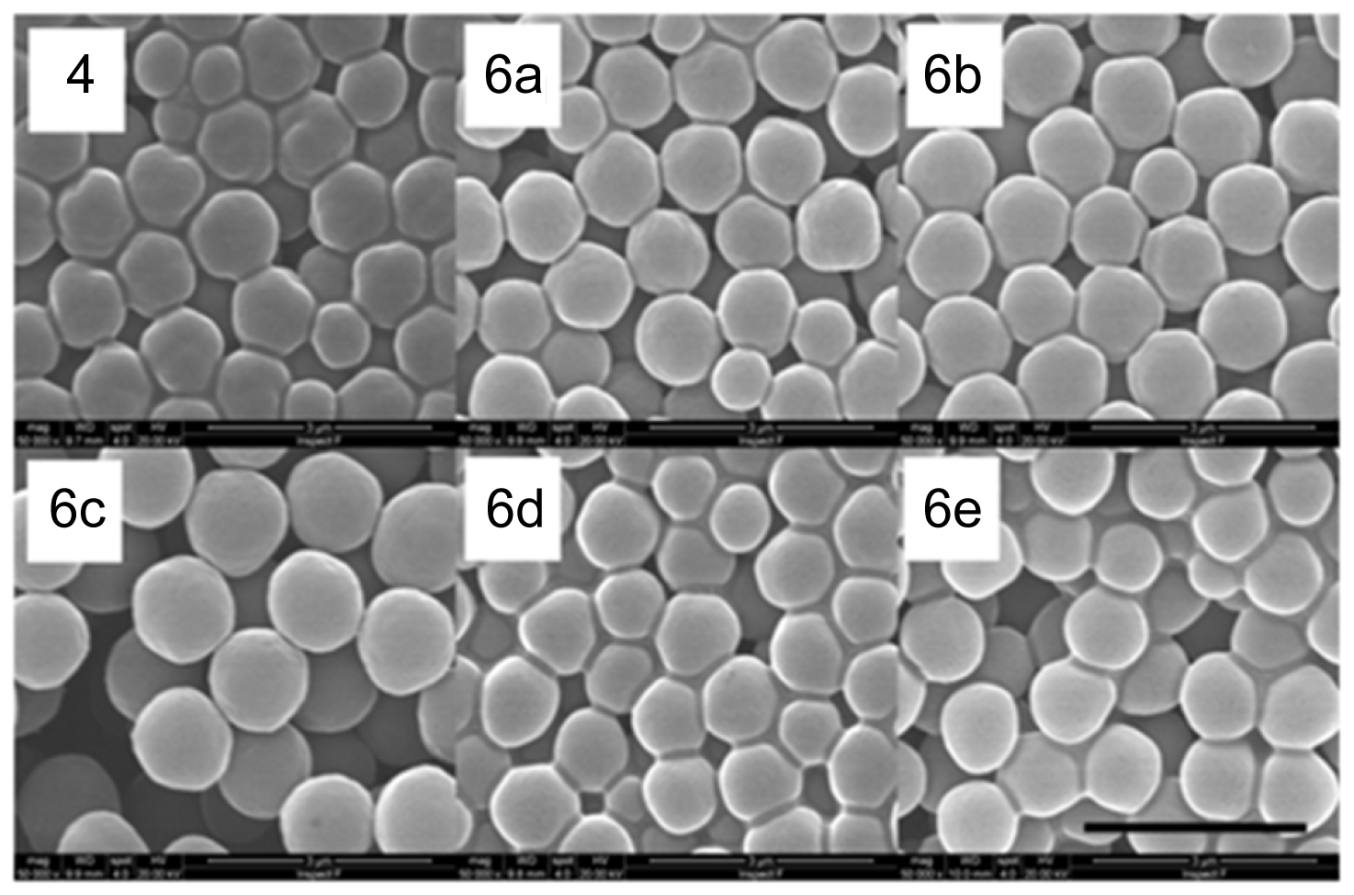
SEM examination of microspheres. SEM images of thiouronium-functionalized microspheres **4**, core-shell microspheres **6a**–**6c** synthesized with varying ratios of co-monomers to seed particles and core shell microspheres **6e** and **6f** synthesized with varying ratios of styrene to methacrylic acid.

To examine whether the proportion of shelling monomer relative to the cores affected shell thickness, a stock solution of co-monomers **1**, **2** and **5** in two-fold, five-fold and ten-fold excesses (by mass, relative to the mass of core particles **4**) was employed in three separate shelling reactions and the size of the resultant microspheres was examined by laser diffractometry (Table 1, **6a**–**c**). Interestingly, a linear relationship between mean particle size and the excess of shelling monomer was established. However, given the magnitude of the standard deviation in particle size, we questioned the relevance of this result and so also examined the microspheres by scanning electron microscopy (SEM). Clearly the particles generated using a ten-fold excess of shelling monomer are larger than the other particles (Figure 2, cf **6c** with **4**, **6a** and **6b**), but it is difficult to discern any further differences in size from these images. Since the SEM showed that all conditions provided a smooth morphology, a two-fold excess of shelling monomer (by mass, relative to the mass of core particles **4**) was selected for use in subsequent experiments.

**Table 1:** Composition and sizing data of microspheres **4** and **6a**–**h.**

Microsphere sample	Co-monomers/core particles^a^	Methacrylic acid/styrene^a^	Mean diameter (µm)	Standard deviation (µm)
**4**	-	-	1.006	0.455
**6a**	2∶1	3∶7	1.013	0.443
**6b**	5∶1	3∶7	1.080	0.444
**6c**	10∶1	3∶7	1.205	0.428
**6d**	2∶1	1∶9	1.052	0.465
**6e**	2∶1	1∶1	1.095	0.345
**6f**	2∶1	3∶7	0.944	0.443
**6g**	2∶1	3∶7	1.08	0.450
**6h**	2∶1	3∶7	0.50	0.320

a ratio determined on the basis of mass

Mean diameter and standard deviation of thiouronium-functionalized microspheres **4** and **4a** and core-shell microspheres **6a**–**h**, as measured dispersed in water using a laser diffractometer.

To evaluate the potential for bioconjugation, the effect of varying the ratio of MAA:styrene in the shelling reaction was examined. Core microspheres **4** were shelled with monomer MAA∶styrene ratios of 1∶9 and 1∶1 and the resultant core-shell microspheres (**6d** and **6e**) were compared with core-shell microspheres **6a** (MAA∶styrene ratio of 3∶7). Examination of microspheres **6d** and **6e** by laser diffractometry and SEM revealed similar sizes (Table 1) and morphology (Figure 2) to microspheres **6a**. The surface carboxyl loading of each sample was then examined by coupling a mono-Fmoc protected diamine to the surface of the particles, *via* an amide bond, and subsequent Fmoc deprotection using 1,8-diazabicyclo[5.4.0]undec-7-ene (DBU) [32] (see Figure S1). As expected, increasing the ratio of MAA∶styrene from 1∶9 (**6d**) to 3∶7 (**6a**) led to an increase in the carboxylic acid surface loading, from 7.7–9.5 µmol g^−1^ to 38.7–61.7 µmol g^−1^. Interestingly however, the microspheres shelled with a 1∶1 ratio of MAA∶styrene (**6e**) showed a markedly reduced surface loading of 10.9–14.5 µmol g^−1^, suggesting that this higher proportion of MAA was not beneficial. A ratio of 3∶7 MMA∶styrene (containing 1% DVB crosslinker) was therefore selected for microsphere shelling in all subsequent experiments.

As well as providing sites for surface conjugation of biomolecules, the carboxyl groups were introduced in order to provide the microspheres with as high a degree of colloidal stability in water as possible. Measurement of the zeta potential of the microspheres, before and after the shelling reaction, was therefore carried out to determine whether the shelling step had conferred a high negative zeta potential upon the microspheres, which would be expected to provide increased electrostatic stabilization. To this end, a fresh batch of core microspheres **4** was synthesized and shelled using our chosen conditions of two fold molar excess of shell co-monomers, with a MAA∶styrene ratio of 3∶7 (containing 1% DVB) to give a second batch of core-shell microspheres **6f**. Sizing data obtained using laser diffractometry showed the size of the resulting core-shell microspheres is reasonably consistent with the previous syntheses (Table 1). As expected, the zeta measurements confirmed that the core microspheres **4** have a low positive zeta potential whilst the core shell microspheres **6f** have a high negative zeta potential (Table 2).

**Table 2:** Zeta potential measurements of microspheres **4** and **6f.**

Microsphere sample	Zeta potential/mV
**4**	+9.86
**6f**	−74.84

Zeta potential measurements, of thiouronium-functionalized microspheres **4** and core–shell microspheres **6f**, determined on the basis of electrophoretic light scattering.

### Orthogonal fluorophore labeling of core and shell

Two further batches of core microspheres **4** were synthesized for evaluation in subsequent core-shell labeling experiments. In order to make core particles of smaller dimension, ethanol∶water mixes of 98∶2 and 95∶5 replaced ethanol in the dispersion polymerization reaction [33]. Carboxyl shells were then grown around the cores of each batch of microspheres using the protocol described above (2-fold excess of co-monomers, by mass, relative to the mass of core particles **4** and a MAA∶styrene ratio of 3∶7). This two step process afforded core-shell microspheres **6g** (98∶2 ethanol∶water) and **6h** (95∶5 ethanol∶water). As demonstrated in Table 1, the addition of 2% water made no difference to the size of the resultant core-shell microspheres 6g (c.f. **6a** and **6f**) whereas 5% water in the dispersion polymerisation reaction effectively halved the diameter of the microspheres. The surface carboxyl loading of these microspheres was also assessed utilizing the Fmoc cleavage assay described above (**6g** = 66.8 µmol g^−1^, **6h** = 57.5 µmol g^−1^).

**Figure 3:**
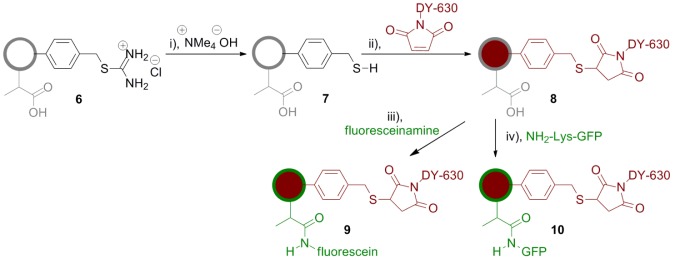
Fluorescent labeling of core-shell microspheres. Reaction conditions: i) DMF, MeOH, RT, 16 h; ii) DMF, RT, 2 h; iii) EDAC, MES pH 5.5, DMF, RT, 18 h; iv)EDAC, MES pH 6.5, NaOH, RT, 2.5 h.

We have previously reported that the thiouronium groups in microspheres **4** can be converted into thiol groups by treatment with base [29]. Accordingly, core shell microspheres **6g** and **6h** were reacted with the relatively hydrophobic base tetramethylammonium hydroxide resulting in the formation of microspheres, **7a** and **7b** respectively, possessing free thiol moieties within their core regions. The orthogonal reactivity of the unmasked thiol groups in the core, relative to the free carboxyl groups in the shell, was then exploited to selectively label the cores of microspheres **7a** and **7b** with maleimide-functionalized DY-630, a far-red dye, to generate fluorescently-labelled microspheres **8a** and **8b**. Moreover, labeling of the cores in this manner proved facile and was accomplished without any cumbersome protection/deprotection steps. Subequently, the bioconjugation potential of the carboxyl-functionalized aqueous-compatible shell was tested by reacting the carboxylic acid groups in the shell with fluoresceinamine under standard EDAC-mediated bioconjugation conditions to generate dual-labeled microspheres **9a** and **9b**. The success of this reaction prompted us to attempt bioconjugation of GFP to microspheres **8a** and **8b** and this approach proved similarly successful giving rise to dual-labelled microspheres **10a** and **10b** respectively (Figure 3). Again it is notable that no additional protection/deprotection steps were required in this second labeling procedure. Moreover both labeling procedures proceeded smoothly in aqueous media demonstrating the hydrophilic nature of the methacrylic acid-shelled microspheres. In this study, DY630 was selected to provide a far-red fluorescence emission that was fully resolvable from the emission of GFP. However, a range of alternative thiol reactive dyes that are a) suitably hydrophobic to be compatible with our labeling strategy and b) have emission maxima across the visible spectrum are commercially available.

### Fluorescence Analysis

**Figure 4:**
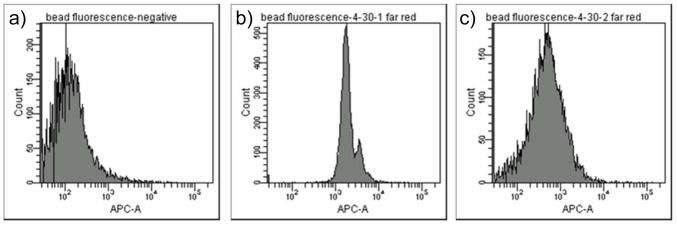
Flow cytometry analysis of microspheres. Flow cytometry analysis of a) unlabelled microspheres **7a** (negative control); b) DY-630 labelled microspheres **8a**; c) DY-630 labelled microspheres **8b**

Following incubation of our microspheres with cells we wished to be able to exploit the inbuilt fluorescence of the microsphere cores to enable us to discriminate between cells that contain microspheres and those that do not. To achieve discrimination in a high throughput manner, we employed fluorescence-activated cell sorting (FACS). Initially, we sought to ascertain whether detection of DY-630 emission from within the cores of the microspheres was possible. An intense fluorescence signal, as measured by FACS, enabled core labeled beads **8a** and **8b** to be both detected and discriminated from unlabeled microspheres **7a**. This study also demonstrated that DY-630 maleimide had successfully been conjugated into the cores of the microspheres. (Figure 4).

**Figure 5:**
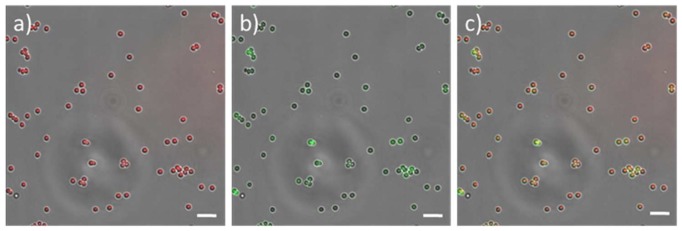
Confocal microscopy of microspheres. Confocal microscope images of DY-630 labelled microspheres conjugated to GFP **10a**: a) excited at 633 nm; b) excited at 488 nm; c) composite image showing co-localization of DY630 and GFP fluorescence. Scale bar = 4 µm.

**Figure 6:**
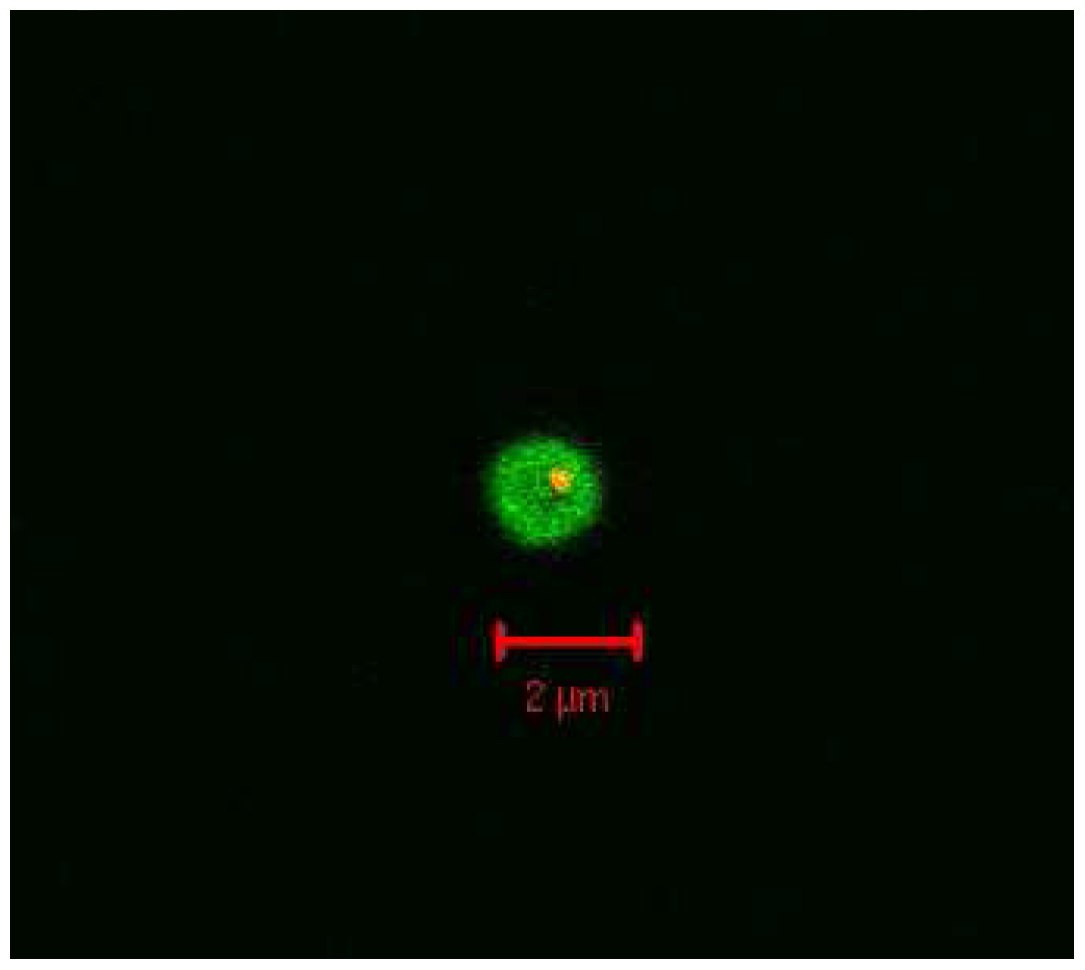
Confocal microscopy of a single microsphere. An overlay confocal microscope image of a DY-630 labelled microsphere conjugated to GFP **10a**: excited at 633 nm (core, red) and at 488 nm (shell, green). Scale bar = 2 µm.

Key to the overall success of these core-shell microspheres is their ability to conjugate biomolecules to the outer shell in the presence of a dye labeled core. As outlined above, this was achieved through the formation of an amide bond between carboxyl groups on the microspheres' **8a** outer shell and pendant amine groups and/or the amino terminus of GFP using standard EDAC coupling conditions. Following conjugation, confocal microscopy demonstrated that far-red fluorescence was spectrally resolvable from the green fluorescence associated with the conjugated protein (Figure 5). Finally, the core-shell architecture of a single microsphere was assessed. Here a close-up overlay confocal microscopy image of a single microsphere clearly shows fluorescence from the shell (green) surrounding that of fluorescence from the core (red, Figure 6).

### Beadfection with mono and dual-labeled microspheres

The microspheres were next assessed for their ability to enter cells by fluorescence-based flow cytometry. This technique allows for the high-throughput quantification of cellular fluorescence and can analyse several thousand cells per second. The technique works by passing live cells in a stream of droplets (where one droplet contains approximately one cell) through a series of lasers, which excite any fluorophore present. Emission from the fluorescent compound is collected by a detector and interpreted by an associated computer program. Previous work had shown that flow cytometry in conjunction with trypan blue, a reagent that is used to quench extracellular fluorescence (from non-internalized microspheres), could be used to establish beadfection efficiency [19]. We wished, if possible, to simplify this uptake efficiency assay. Accordingly, we incubated samples of HeLa cells with our microspheres for twelve hours and assessed microsphere uptake by the cells using flow cytometry alone and compared the results obtained with data from flow cytometry analysis that also employed trypan blue. No appreciable difference was observed in the data relating to the efficiency of microsphere uptake by the cells after 12 hours or more incubation time (Figure S1). This preliminary study demonstrated that flow cytometry, without trypan blue, enables effective selection of cells with internalized microspheres. Accordingly, this simplified procedure was employed in all subsequent cellular uptake experiments.

**Figure 7:**
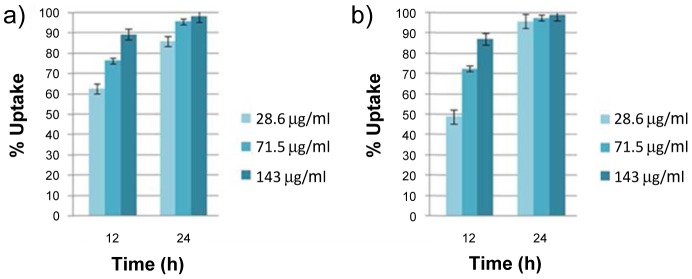
Cellular uptake of core-labeled microspheres, assessed by FACS. Percentage cellular uptake of DY-630 labelled microspheres **8** by HeLa cells, as measured by flow cytometry: a) **8a** (1 µm diameter); b) **8b** (500 nm diameter).

To establish the effect of microsphere concentration upon beadfection efficiency, DY-630 core-labelled microspheres **8a** and **8b** were assessed for uptake over 12 and 24 hours in HeLa cells at a microsphere concentration ranging from 28.6–143 µg/mL using quantitative fluorescence-based flow cytometry (Figure 7). Control measurements were carried out on both the fluorescent microspheres alone, and on the HeLa cells prior to incubation with microspheres, in order to determine the respective size range and fluorescence profiles of each. Only fluorescence emitted from particles within the size range corresponding to HeLa cells was used to calculate cellular uptake of the fluorescent microspheres, so as to avoid inaccuracies arising due to measurement of fluorescence from any microspheres remaining in the cell media. Furthermore, only cells that showed fluorescence in excess of control cells (incubated in the absence of microspheres) were considered to have successfully taken up microspheres. For the 1 µm microspheres **8a**, uptake was found to be maximal (98%) at the higher concentration (143 µg/mL) and after 24 hours incubation time. However, uptake was found to be high (88%) even after a shorter 12 hour incubation time showing the efficient and rapid translocation of microspheres into cells. The uptake profile was very similar for the intermediate microsphere concentration (71.5 µg/mL) although here, an incubation time of 24 hours was required to obtain very high (95%) uptake efficiency. Virtually identical uptake efficiency profiles were observed for the smaller 500 nm microspheres **8b**, which again gave excellent uptake efficiency (99%) at the higher concentration (143 µg/mL) following a 24 hour incubation period (Figure 7).

**Figure 8:**
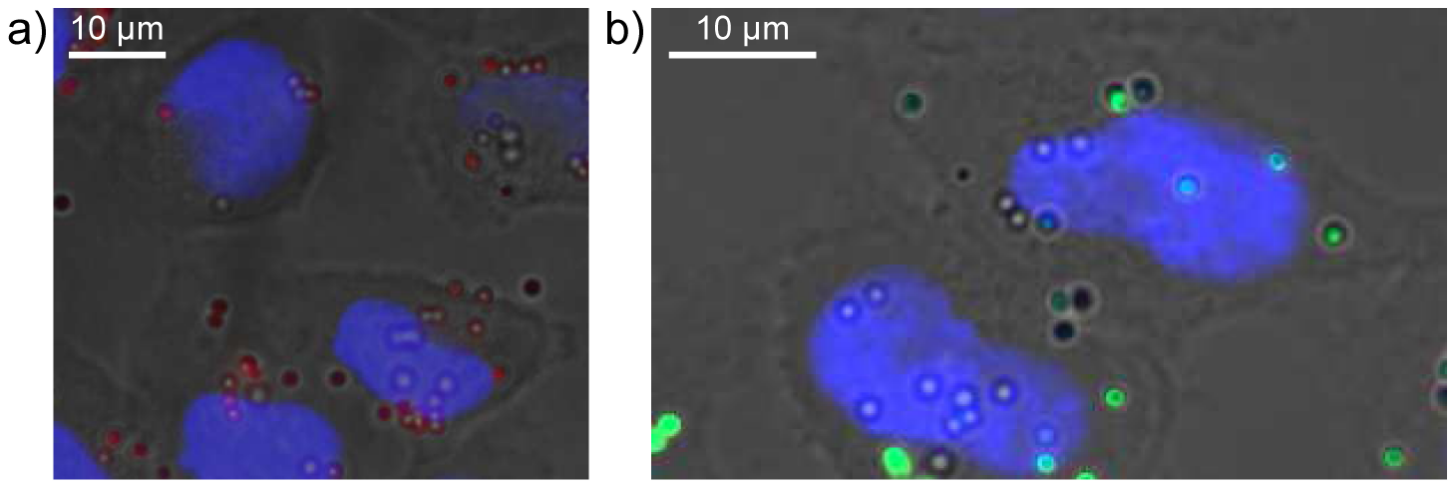
Fluorescent microscopy of beadfected HeLa cells. Fluorescence microscope images obtained from a Z stack set of images (approximately 0.5 µm z-steps, taken from the top to the bottom focal plane of the cells, 10–12 slices in total) of HeLa cells beadfected with a sample of DY-630-labelled microspheres conjugated via their shells to GFP 10a: a) under irradiation at 633 nm to show DY-630 fluorescence; b) under irradiation at 433 nm to show GFP fluorescence. In each case the cells have been fixed and their nuclei stained with 4',6-diamidino-2-phenylindole (DAPI). Scale bar = 10 µm.

**Figure 9:**
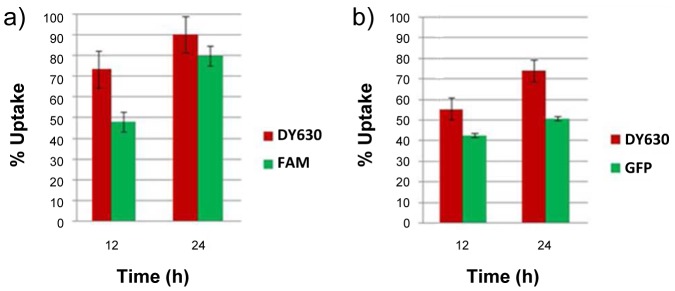
Ceullular uptake of dual core/shell-labeled microspheres, assessed by FACS. Cellular uptake of shell-conjugated, DY-630 core-labelled microspheres by HeLa cells, as measured by flow cytometry: a) shell conjugated to fluoresceinamine 9a; b) shell conjugated to GFP 10a.

The orthogonal nature of our dual functionalized microspheres means that they have the potential to allow for the independent tracking of a delivery vehicle and its cargo. To evaluate this potential we investigated the cellular uptake of DY630 core-labelled 1 µm microspheres **8a** whose shells had been conjugated (*via* EDAC coupling, Figure 3) either to fluoresceinamine **9a** or GFP **10a** (Figure 8). Since the study assessing the uptake of core-labelled microspheres **8a** and **8b** had shown that high beadfection efficiencies could be achieved at microsphere concentrations of 71.5 and 143 µg/mL, cellular uptake was assessed at an intermediate concentration level of 86 µg/mL. As before, incubation was carried out for periods of 12 and 24 hours and beadfection efficiency was assessed by flow cytometry (Figure 9). In each case, the fluorescence assessed via the core label (DY630) gave higher uptake values than when the same cells were assessed via the shell-conjugated fluor (fluoresceinamine or GFP). We ascribe this difference to the more isolated environment within the core of the microspheres rendering the fluorescent label less prone to quenching effects, which may occur at the cell-microsphere interface (*i.e*. the microsphere shell). This difference notwithstanding, our results indicate that it is indeed possible to beadfect cells with dual-labelled one micron-sized particles and still obtain reasonably good (50%, in the case of a protein cargo conjugated to the shell) and good (90%, in the case of a small molecule cargo conjugated to the shell) uptake efficiencies.

Finally, delivery of active biomolecules into cells would be of little value if the delivery method had a deleterious effect on the viability of the cells themselves. We therefore carried out 3-(4,5-dimethylthiazol-2-yl)-2,5-diphenyltetrazolium bromide (MTT) assays [19] on HeLa cells following uptake of either the 1 µm (**10a**) or 500 nm (**10b**), DY630 labeled microspheres conjugated to GFP (initial bead concentrations of 43, 86 and 172 µg/mL). These studies indicated that even at the highest concentrations for both bead sizes, 92% of the cells remained viable following an incubation period of 24 hours. This finding is in agreement with earlier studies that confirm, via MTT assays, that cells remain viable 24 hours after beadfection [19], [23]–[27]. Clearly, MTT assays (which assess a population rather than individual cells) has increasingly questionable relevance over longer time frames since the microspheres become diluted within a population as the cells divide; this means that viability data could result from new, non-beadfected cells after division. However, alternative evidence for longer-term, post-beadfection viability is compelling. For example, E14tg2a embryonic stem cells, beadfected with microspheres carrying a β-galactosidase cargo, retain the ability to process vital β-galactosidase substrates 3 days after beadfection [34], whilst gene expression analysis showed that beadfection had no significant effect on HEK293T and L929 cells even after incubation periods of two days [19]. Such time periods will be suitable for most in vitro assays, but in the much longer term, beadfected stem cells not only remain viable, but their ability to differentiate is unaffected by the presence of microspheres up to 30 days post beadfection [35].

### Conclusions

In summary, we have developed bio-compatible, core-shell polymer microspheres for use in *in vitro* bioimaging and biomolecule delivery. Labeling of the thiolated core with thiol reactive maleimide dyes is fully orthoganol with conjugation of bio-molecules to the outer carboxyl-functionalized shell. We have also used HeLa cells as a model system to demonstrate the feasibility of using our dual functionalized microspheres as a cellular delivery system for proteins and other biologically relevant molecules, and that their cellular delivery efficiency can be monitored *via* an internal (core) fluorescent label. Futhermore, we have shown that our microsphere-based delivery system does not have a significant deleterious effect on cell viability.

## Materials and Methods

### General Considerations

Chemical reagents were purchased from Aldrich and used as received, except for styrene **1** which was washed with an aqueous solution of NaOH (1 M) and dried over magnesium sulfate prior to polymerization. ^1^H and ^13^C NMR spectroscopy was carried out using a Bruker Avance 300 spectrometer. Chemical shifts are measured in ppm, and multiplicity is denoted as follows: s = singlet, d = doublet, dd = double doublet. Sizing data for polystyrene microspheres was obtained using a Sympatec Helos Particle Size Analyser. Zeta potential measurements were acquired using a Beckmann Coulter Delta Nano C Particle Analyzer which was equipped with Delta Nano 2.1 software and a Delta Nano C Zeta Potential flow cell. Samples were measured in a Delta Nano C Zeta Potential flow cell and measurements were repeated five times and an average value determined. Scanning electron microscopy (SEM) images were obtained using a FEI Inspect F FEG instrument operating at 20 kV. All samples were sputter-coated with a thin overlayer of gold prior to inspection to prevent sample-charging effects. Unless otherwise stated all cell culture media reagents were obtained from PAA (UK). Nunc Lab-Tek 4 well chamber slides were obtained from Fisher Scientific (UK).

### Synthesis of 4-vinylbenzyl isothiouronium chloride (4-VBTU) 3

4-VBTU **3** was synthesized by a modification of the method reported by Nelson Jr. [36] as described previously (Figure 1) [29]. ^1^H NMR (300 MHz, D_2_O) δ 4.39 (s), 5.33 (d, *J* = 11.7 Hz), 5.86 (d, *J* = 18.3 Hz), 6.78 (dd, *J* = 11.1 Hz and 11.1 Hz), 7.43 (d, *J* = 8.1 Hz), 7.51 (d, *J* = 8.4 Hz); ^13^C NMR (75 MHz, D_2_O) 35.8, 115.0, 126.7, 129.2, 133.7, 135.9

### Synthesis of thiouronium microspheres 4

Thiouronium microspheres **4** were synthesized as described previously [29]. All core particles were synthesized using 1.0 mol.% of 4-VBTU **3** relative to styrene **1**. Core particles **4** were synthesized in 100% absolute ethanol. Smaller core particles for core-shell microspheres **6g** and **6h** were synthesized in ethanol/water; 98∶2 and 95∶5 respectively.

### Seeded emulsion polymerization with thiouronium microspheres 4

The following general procedure describes the appropriate amounts of co-monomers styrene **1** and methacrylic acid (MAA) **5** for the synthesis of core-shell microspheres **6a**, **6d**, and **6e**–**h**; ratio of co-monomers/microspheres = 2∶1 by mass. In order to vary the shell thickness (**6b** and **6c**), the amounts of styrene **1** and MAA **5** were changed so that this ratio equaled 5∶1 or 10∶1, while the amounts of all other chemicals were kept constant. The mass/mass ratio of MAA **5**/styrene **1** was also kept constant at 3∶7, except for microspheres **6d** and **6e** (MAA **5**/styrene **1** equaled 1∶9 and 1∶1 respectively). Sodium dodecyl sulfate (SDS) (4.0 mg, 1.38×10^−2^ mmol) was dissolved in water (4.80 mL) and thiol microspheres **4** (100 mg) were resuspended in this aqueous solution. AIBN (2 mg, 1.21×10^−2^ mmol) was dissolved in styrene **1** (154 µL, 1.34 mmol), to which DVB **2** (10.9 µL, 7.65×10^−2^ mmol), MAA **5** (59.1 µL, 6.97×10^−1^ mmol) and hexadecane (25.8 µL, 8.81×10^−2^ mmol) were added. This monomer mixture was added to an aqueous suspension of thiouronium microspheres **4**, with stirring, at room temperature and the reaction mixture was degassed by bubbling with nitrogen at room temperature for 15 minutes. The temperature was then increased to 70°C and stirring was continued for 5 hours. The reaction mixture was cooled to room temperature and filtered through a pad of glass wool to separate any coagulum formed from the polymer microspheres in suspension. Carboxyl-shelled/thiouronium-core microspheres **6** were isolated by centrifugation (15 minutes, 6000 rpm), decantation of the supernatant and were purified with several wash/centrifugation cycles: water (2×5 mL), methanol (2×5 mL), water (2×5 mL) and were finally resuspended in water (5 mL).

### Synthesis of thiol-functionalized microspheres 7

Carboxyl-shelled/thiouronium-core microspheres **6** (200 mg) were washed with DMF (2×5 mL), with centrifugation (5 minutes, 6000 rpm) and decantation of the supernatant after each wash, and then resuspended in DMF (5 mL). Tetramethylammonium hydroxide solution (25 wt.% in methanol, 100 µL) was added to the reaction mixture, which was then shaken at room temperature for 16 hours. The polymer microspheres were isolated by centrifugation (5 minutes, 6000 rpm) and decantation of the supernatant. Washing/centrifugation cycles were carried out as above: DMF (2×5 mL), methanol (2×5 mL) and water (2×5 mL). The resultant carboxyl-shelled/thiol-core microspheres **7** were finally resuspended in water (5 mL).

### Fmoc Loading Assay

Core/shell microspheres in water (approx 10.0 mg of microspheres) were isolated by centrifugation (1 min, 12000 rpm) and decantation of the supernatant. The microspheres were subjected to wash/centrifugation cycles with DMF (2×2 mL) and were then resuspended in DMF (1 mL). Fmoc-1,3-diaminopropane hydrochloride (3.39×10^−2^ mmol) was dissolved in DMF (1 mL) with *O*-(benzotriazol-1-yl)-*N*,*N*,*N*′,*N*′-tetramethyluronium tetrafluoroborate (TBTU, 13.0 mg, 4.05×10^−2^ mmol) and *N*-ethyldi*iso*propylamine (DIPEA, 7.2 µL, 7.47×10^−2^ mmol) and this solution was added to the suspension. The reaction mixture was shaken at room temperature for 1 hour and the surface modified microspheres were isolated by centrifugation (1 min, 12000 rpm) and decantation of the supernatant. Excess reagents were removed by repeated wash/centrifugation cycles with DMF (5×2 mL), followed by methanol (2×5 mL) and the microspheres were then dried in vacuo in order to accurately determine their mass. The microspheres were subsequently resuspended in a solution of 1,8-diazabicyclo[5.4.0]undec-7-ene (DBU, 20.0 µL) in DMF (1 mL) and were shaken at room temperature for 20 minutes. The reaction mixture was then transferred to a volumetric flask and acetonitrile was added until the total volume was equal to 5 mL. In order to remove the resultant microspheres, this suspension was centrifuged (15 mins, 8000 rpm) and the supernatant was then separated by decantation and collected. A UV/vis spectrometer was then used to measure the absorbance of the signals at 294 and 305 nm in the supernatant solution, which corresponded to the amount of a Fmoc derivative which had been cleaved from the surface of the microspheres.

### Calculation of extinction coefficient for Fmoc loading assay

Fmoc-1,3-diaminopropane hydrochloride (10 mg, 1.69×10^−2^ mmol) was dissolved in DMF (2 mL) with DBU (40 µL) and the solution was shaken at room temperature for 20 minutes. Acetonitrile was then added to the solution until the total volume was equal to 10 mL. Four serial dilutions were made, each time adding 5 mL of acetonitrile to 5 mL of the preceding solution. A solution was also prepared by dissolving DBU (20 µL) in DMF (1 mL) and adding acetonitrile until the total volume was equal to 5 mL and this was used to take a background reference measurement on the UV/vis spectrometer prior to measuring the absorbance of the serial dilutions. For each serial dilution, the absorbance of characteristic Fmoc signals at 294 and 305 nm was measured and these values were plotted as a function of the concentration of each solution. A trend line was plotted for each of these linear correlations, and the gradient of each line was taken to be the extinction coefficient, ε, for the corresponding wavelength. Following conjugation of Fmoc-1,3-diaminopropane hydrochloride to each sample of core-shell microspheres and subsequent Fmoc cleavage with DBU (see Fmoc Loading Assay, above), the absorbance at 294 and 304 nm was measured for each cleavage solution. This allowed for quantification of the carboxyl loading (mmol g^−1^) of each sample of microspheres, using the following equation:

Carboxyl loading (mmol g^−1^) = [(Abs/ε)*V]/mass of microspheres

Values for the carboxyl loading measured based on absorbance at 294 nm and based on the absorbance at 304 nm were then averaged to give the final recorded carboxyl loading ( µmol g^−1^).

### Labeling the thiol cores of core-shell microspheres 6 with DY-630 maleimide

Microspheres **6** (5.0 mg) were washed with DMF (2×1 mL), with centrifugation (5 minutes, 6000 rpm) and decantation of the supernatant after each wash, and then resuspended in DMF (1 mL). A solution of DY630 maleimide (Dyomics GmbH) in DMF (1 mg mL^−1^, 50 µL, 1.32×10^−5^ mmol) was added to the suspension of microspheres, which was then shaken at room temperature for 2 hours. Fluorescent core-labelled microspheres **8** were isolated by centrifugation (2 minutes, 6000 rpm), decantation of the supernatant and were purified with several wash/centrifugation cycles: DMF (5×1 mL), water (2×1 mL) and were finally resuspended in water (1 mL).

### Conjugation of fluoresceinamine (FAM) onto the shell of DY-630 core-labelled microspheres 8

DY630 core-labelled microspheres **8** (5.0 mg) were washed with 2-(*N*-morpholino)ethanesulfonic acid (MES, pH 5.5, 3×1 mL) with centrifugation (5 minutes, 6000 rpm) and decantation of the supernatant after each wash, before activation with *N*-ethyl-*N*'-(3-dimethylaminopropyl)-carbodiimide.HCl (EDAC, 3.5 mg, 1.8×10^−2^ mmol) in MES (1 mL, pH 5.5) for 2 hours at 25°C. After this time, the beads were obtained by centrifugation as previously and FAM (1.2 mg, 3.5×10^−3^ mmol) was added in DMF. The reaction was allowed to continue for 18 hours at 25°C, before washing sequentially with DMF (3×1 mL), methanol (3×1 mL) and water (3×1 mL). The dual-labelled microspheres **9** were stored in water at 4°C.

### Bioconjugation of green fluorescent protein (GFP) onto the shell of DY-630 core-labelled microspheres 8

GFP (with a His-tag at the amino terminus) was expressed from tuner DE3™ bacteria and purified by affinity chromatography through a nickel-agarose column. The purified GFP was then dialysed into MES buffer (50 mM, pH 6.0) and the solution was diluted to a GFP concentration of 10 mg mL^−1^. DY630 core-labeled microspheres **8** (1.0 mg) were resuspended in MES buffer (100 µL, 50 mM, pH 6.0) followed by the addition of 300 µL of the 10 mg mL^−1^ GFP/MES solution. This suspension was incubated with rolling at room temperature for 15 minutes. Following this incubation period, EDAC (0.4 mg, 2.1×10^−3^ mmol) in MES (50 µL, 50 mM, pH 6.0) was added to the aqueous suspension and the pH was adjusted to 6.5 by addition of aqueous sodium hydroxide (0.5 M). The reaction mixture was then incubated, with rolling at room temperature, for a further 2.5 hours then centrifuged (5 minutes, 8000 rpm) and the supernatant, containing excess GFP was removed by decanting. The bead pellet was resuspended in sodium phosphate buffer solution (500 µL, 100 mM) by vortexing and was then centrifuged (5 minutes, 8000 rpm) and the supernatant was decanted off. This washing procedure was repeated for a further cycle and the bioconjugated microspheres **10** were finally resuspended in sodium phosphate buffer (500 µL, 100 mM).

### “Beadfection” of HeLa cells for flow cytometric analysis

Human cervical cancer (HeLa) cells were cultured at 37°C under 5% CO_2_ until 70–80% confluency in Roswell Park Memorial Institute Medium (RPMI-1640) supplemented with 10% fetal bovine serum (FBS), 100 units/mL penicillin/streptomycin and 4 mM L-glutamine. Cells were obtained for experiments by gentle washing with phosphate buffered saline (PBS, pH 7.4), harvesting with trypsin/EDTA at 37 °C and collection of the cell pellet by centrifugation at 1100 rpm for 4 minutes. Cells were resuspended to a concentration of 8.6×10^4^ cells/mL in growth medium and seeded to a 24-well plate. After 24 hours at 37°C under 5% CO_2_, the old media was removed and replaced with fresh media (350 µL) containing microspheres at concentrations ranging from 28.6–143 µg/mL. After 6, 12 or 24 hours, the bead media was removed and the cells were washed in PBS (2×200 µL) and the cells harvested *via* trypsination as described above. For flow cytometry experiments the cell pellet was resuspended in 2% FBS in PBS (300 µL) and analysis was made on a BD Bioscience FACS Aria and assessed using FACS Diva software. Excitation of DY630 was made with a 633 nm JDS Uniphase HeNe air-cooled laser and emission collected using a 660/20 band pass filter. Fluorescein was excited with a 488 nm Coherent Sapphire solid state laser and emission collected using a 530/30 band pass filter. The fluorescent microspheres alone in PBS, as well as HeLa cells incubated in the absence of microspheres, were used as controls to determine the size range and fluorescence profiles of the beads and cells. During the flow cytometric analysis of cells incubated in the presence of microspheres, cells were gated to select only those that appeared in the correct size range of the cells and that exceeded the fluorescence of non-beadfected control cells (*i.e*. only the fluorescence from cell-internalized fluorescent microspheres was considered and the fluorescence from non-internalized microspheres and non-beadfected cells in the media was ignored).

### Zeta potential measurements

A sample of microspheres (1 mg) was suspended in deionised water (1 ml) by ultrasonication, which was then injected into a Delta Nano C Zeta Potential flow cell using a 1 ml syringe. The flow cell has a capacity of 0.7 ml, surplus sample was added to ensure that the cell was devoid of air. Measurements were repeated five times and an average value determined.

### MTT Assays

HeLa cells were cultured as described above. They were subsequently seeded to a 96-well plate at a density of 1×10^3^ cells/well in growth media following harvesting by trypsination and collection of the cell pellet by centrifugation. Microspheres were added in fresh phenol-red free growth media at concentrations of 28.6–143 µg/mL. After 24 hours, 3-(4,5-dimethylthiazol-2-yl)-2,5-diphenyltetrazolium bromide (MTT) was added in phenol red-free media (5 mg/mL, 10 µL per well) and the cells incubated for a further 5 hours. After this time, MTT solubilization solution was added (10% Triton-X, 0.1 N HCl in *iso*propanol, 100 µL) to dissolve formazan crystals and the well plate was gently shaken overnight. Analysis of absorbance was made at 570 nm. Untreated cells were considered 100% viable.

### “Beadfection” of HeLa cells for fluorescent microscopy

HeLa cells were maintained in Dulbecco's modified Eagle's medium (DMEM) supplemented with 10% Fetal Bovine serum, 2 mM L-glutamate, 100 units/ml penicillin and 100 µg/ml Streptomycin. The cells were obtained for experiments by washing in Dulbecco's Phosphate Buffered Saline (D-PBS) (Gibco), harvesting with trypsin/EDTA and the cells were then collected by centrifugation (1100 rpm, 4 minutes). Cells were resuspended in 10 ml DMEM media and 5.6×10^4^ cells were used to seed each well of the chamber slide in a total volume of 250 µl media. After 24 hours the media was replaced with media containing the GFP-conjugated microspheres at a final concentration of 50 µg ml^−1^. After 48 hours the cells were washed twice with 250 µl of DMEM, and 250 µl of fresh media was added to each well. Images were obtained using a Zeiss Axiovert 200 M fluorescent microscope (100× objective) fitted with a Hamamatsu CCD camera, driven by Velocity 4.2.1 software (Improvision UK) and equipped with a ASI Z stage (Zeiss). DAPI fluorescence was detected using Zeiss filter set 2 (ex 365 nm, em LP 420 nm); GFP fluorescence was detected using the Zeiss filter set 10 (ex. 450–490 nm, em. 515–565 nm); and DY-630 fluorescence was detected using Zeiss filter set 15 (ex 546/12 nm, em LP 590 nm).

## Supporting Information

Figure S1 **Flow cytometry data expressed in the form of % fluorescent cells versus time for flow cytometry in fetal bovine serum (FBS)/phosphate-buffered saline (PBS) and 0.2% trypan blue in Hanks' Balanced Salt Solution (HBSS).**
(TIF)

## References

[1] Pallen MJ (2011) Time to recognise that mitochondria are bacteria? Trends Microbiol 19: 58–64. 21123072 10.1016/j.tim.2010.11.001

[2] Kutschera U, Niklas KJ (2005) Endosymbiosis, cell evolution, and speciation. Theory in Biosciences 124: 1–24. 17046345 10.1016/j.thbio.2005.04.001

[3] Wang SH, Lee CW, Chiou A, Wei PK (2011) Size-dependent endocytosis of gold nanoparticles studied by three-dimensional mapping of plasmonic scattering images. J Nanobiotechnol 8 Article 33. 10.1186/1477-3155-8-33 PMC3236302 21167077

[4] Dhar S, Daniel WL, Giljohann DA, Mirkin CA, Lippard SJ (2009) Polyvalent oligonucleotide gold nanoparticle conjugates as delivery vehicles for platinum(IV) warheads. J Am Chem Soc 131: 14652–14653. 19778015 10.1021/ja9071282 PMC2761975

[5] Li X, Xie QR, Zhang J, Xia W, Gu H (2011) The packaging of siRNA within the mesoporous structure of silica nanoparticles. Biomaterials 32: 9546–9556. 21906804 10.1016/j.biomaterials.2011.08.068

[6] Gao XH, CuiYY, Levenson RM, Chung LWK, Nie SM (2004) In vivo cancer targeting and imaging with semiconductor quantum dots. Nat Biotechnol 22: 969–976. 15258594 10.1038/nbt994

[7] Petkau K, Kaeser A, Fischer I, Brunsveld L, Schenning APHJ (2011) Pre- and postfunctionalized self-assembled π-conjugated fluorescent organic nanoparticles for dual targeting. J Am Chem Soc 133: 17063–17071. 21913650 10.1021/ja2075345

[8] Scutaru AM, Krüger M, Wenzel M, Richter J, Gust R (2011) Investigations on the use of fluorescence dyes for labeling dendrimers: cytotoxicity, accumulation kinetics, and intracellular distribution. Bioconjug Chem 21: 2222–2226. 10.1021/bc1001906 21049938

[9] Wang JT, Wang J, Han JJ (2011) Fabrication of advanced particles and particle-based materials assisted by droplet-based microfluidics. Small 7: 1728–1754. 21618428 10.1002/smll.201001913

[10] Adami RC, Seth S, Harvie P, Johns R, Fam R, et al (2011) An amino acid-based amphoteric liposomal delivery system for systemic administration of siRNA. Molecular Therapy 19: 1141–1151. 21505423 10.1038/mt.2011.56 PMC3129796

[11] Higuchi Y, Wu C, Chang KL, Irie K, Kawakami S, et al (2011) Polyamidoamine dendrimer-conjugated quantum dots for efficient labelling of primary cultured mesenchymal stem cells. Biomaterials 32: 6676–6682. 21700331 10.1016/j.biomaterials.2011.05.076

[12] Algar WR, Prasuhn DE, Stewart MH, Jennings TL, Blanco-Canosa JB, et al (2011) The controlled display of biomolecules on nanoparticles: A challenge suited to bioorthogonal chemistry. Bioconjug Chem 22: 825–858. 21585205 10.1021/bc200065z

[13] Braeckmans K, Buyens K, Naeye B, Vercauteren D, Deschout H, et al (2010) Advanced fluorescence microscopy methods illuminate the transfection pathway of nucleic acid nanoparticles. J Control Release 148: 69–74. 20833214 10.1016/j.jconrel.2010.08.029

[14] Tantra R, Knight A (2011) Cellular uptake and intracellular fate of engineered nanoparticles: A review on the application of imaging techniques. Nanotoxicol 5: 381–392. 10.3109/17435390.2010.512987 20846020

[15] Fubini B, Fenoglio I, Tomatis M, Turci F (2011) Effect of chemical composition and state of the surface on the toxic response to high aspect ratio nanomaterials. Nanomedicine (Lond) 6: 899–920. 21793679 10.2217/nnm.11.80

[16] Zhao E, Zhao Z, Wang J, Yang C, Chen C, et al (2012) Surface engineering of gold nanoparticles for in vitro siRNA delivery. Nanoscale 4: 5102–5109. 22782309 10.1039/c2nr31290e

[17] Dominska M, Dykxhoorn DM (2010) Breaking down the barriers: siRNA delivery and endosome escape. J Cell Sci 123: 1183–1189. 20356929 10.1242/jcs.066399

[18] Steinkamp JA, Wilson JS, Saunders GC, Stewart C (1982) Phagocytosis: flow cytometric quantitation with fluorescent microspheres. Science 215: 64–66. 7053559 10.1126/science.7053559

[19] Alexander L, Pernagallo S, Livigni A, Sanchez-Martin RM, Brickman JM, et al (2010) Investigation of microsphere-mediated cellular delivery by chemical, microscopic and gene expression analysis. Mol BioSyst 6: 399–409. 20094660 10.1039/b914428e

[20] Reetz MT, Carsten JR, Dröge MJ, Quax WJ (2002) Immobilization of chiral enzyme inhibitors on solid supports by amide-forming coupling and olefin metathesis. Tetrahedron 58: 8465–8473.

[21] Slomkowski S, Basinka T, Miska B (2002) New types of microspheres and microsphere-related materials for medical diagnostics. Polym Adv Technol 13: 906–918.

[22] Bousalem S, Benabderrahmane S, Sang YYC, Mangeney C, Chehimi MM (2005) Covalent immobilization of human serum albumin onto reactive polypyrrole-coated polystyrene latex particles. J Mater Chem 15: 3109–3116.

[23] Bradley M, Alexander L, Duncan K, Chennaoui M, Jones AC, et al (2008) pH sensing in living cells using fluorescent microspheres. Bioorg Med Chem Lett 18: 313–317. 17988866 10.1016/j.bmcl.2007.10.075

[24] Sánchez-Martín RM, Cuttle M, Mittoo S, Bradley M (2006) Microsphere-based real-time calcium sensing. Angew Chem Int Ed 45: 5472–5474. 10.1002/anie.200601242 16847849

[25] Sánchez-Martín RM, Muzerelle M, Chitkul N, How SE, Mittoo S, et al (2005) Bead-based cellular analysis, sorting and multiplexing. ChemBioChem 6: 1341–1345. 15973759 10.1002/cbic.200500059

[26] Sánchez-Martin RM, Alexander L, Muzerelle M, Cardenas-Maestre JM, Tsakiridis A, et al (2009) Microsphere-mediated protein delivery into cells. ChemBioChem 10: 1453–1456. 19444829 10.1002/cbic.200900136

[27] Alexander LM, Sanchez-Martin RM, Bradley M (2009) Knocking (anti)-sense into cells: The microsphere approach to gene silencing. Bioconjug Chem 20: 422–426. 19245252 10.1021/bc800529r

[28] Vagner J, Barany G, Lam KS, Krchnak V, Sepetov NF, et al (1996) Enzyme-mediated spatial segregation on individual polymeric support beads: Application to generation and screening of encoded combinatorial libraries. Proc Natl Acad Sci USA 93: 8194–8199. 8710846 10.1073/pnas.93.16.8194 PMC38645

[29] Behrendt JM, Afzaal M, Alexander LM, Bradley M, Hine AV, et al (2009) Thiol-containing microspheres as polymeric ligands for the immobilisation of quantum dots. J Mater Chem 19: 215–221.

[30] Jose AJ, Ogawa S, Bradley M (2005) Tuning the pore size and surface area of monodisperse poly(methyl acrylate) beads via parallel seeded polymerisation. Polymer 46: 2880–2888.

[31] Kobayashi K, Senna M (1992) Independent control of mechanical and chemical properties of monodispersed polystyrene–divinyl benzene microspheres by two-step polymerization. J Appl Polym Sci 46: 27–40.

[32] Gude M, Ryf J, White PD (2002) An accurate method for the quantitation of Fmoc-derivatized solid phase supports. Lett Pept Sci 9: 203–206.

[33] Paine AJ, Luymes W, McNulty J (1990) Dispersion polymerization of styrene in polar solvents. 6. Influence of reaction parameters on particle size and molecular weight in poly(N-vinylpyrrolidone)-stabilized reactions. Macromol 23: 3104–3109.

[34] Tsakiridis A, Alexander LM, Gennet N, Sanchez-Martin RM, Livignia A, et al (2009) Microsphere-based tracing and molecular delivery in embryonic stem cells. Biomaterials 30: 5853–5861. 19608269 10.1016/j.biomaterials.2009.06.024

[35] Gennet N, Alexander LM, Sanchez-Martın RM, Behrendt JM, Sutherland AJ, et al (2009) Microspheres as a vehicle for biomolecule delivery to neural stem cells. New Biotechnol 25: 442–449. 10.1016/j.nbt.2009.05.006 19524076

[36] Nelson Jr SJ (1966) S-(4-Vinylbenzyl)-isothiourea and isothiouronium chloride. United States Patent 3260748.

