# Supplementary figures and images for: Synthesis and Characterization of Dual-Functionalized Core-Shell Fluorescent Microspheres for Bioconjugation and Cellular Delivery
Source: PLoS One. 2013 Mar 19;8(3):e50713. doi: 10.1371/journal.pone.0050713 (PMC3602537; doi:10.1371/journal.pone.0050713)

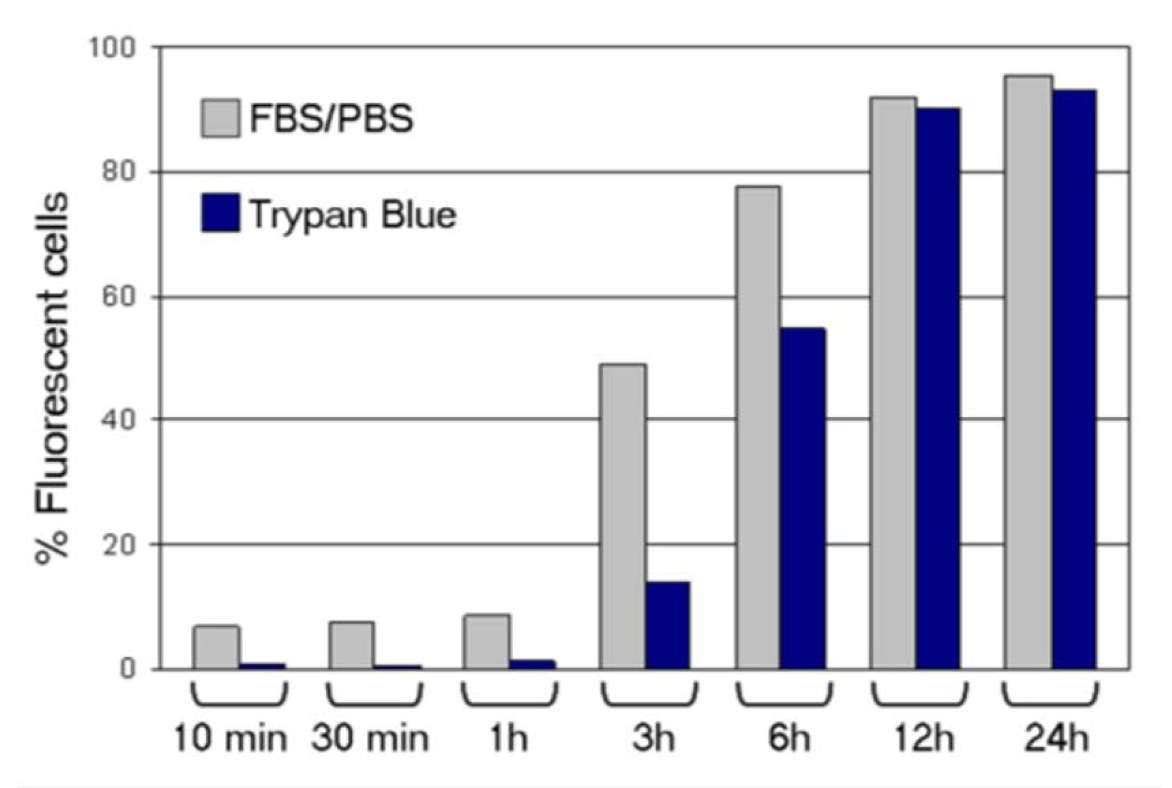

Supplement: Figure S1 — Flow cytometry data expressed in the form of % fluorescent cells versus time for flow cytometry in fetal bovine serum (FBS)/phosphate-buffered saline (PBS) and 0.2% trypan blue in Hanks' Balanced Salt Solution (HBSS). (TIF) [file pone.0050713.s001.tif]
